# Clinical and genetic landscape of neuronopathic gaucher disease in Ukraine: hepatosplenomegaly and diagnostic delay

**DOI:** 10.1186/s13023-026-04578-x

**Published:** 2026-08-31

**Authors:** Nataliia Samonenko, Nataliia Olkhovych, Olena Okhotnikova, Nataliia Gorovenko

**Affiliations:** 1 Shupyk National Healthcare University of Ukraine, Kyiv, Ukraine; 2 National Children’s Specialized Hospital “Okhmatdyt”, Kyiv, Ukraine; 3 National Scientific Center “Institute of Cardiology, Clinical and Regenerative Medicine Named After M.D. Strazheska”, Kyiv, Ukraine

**Keywords:** Gaucher disease, Neuronopathic gaucher disease, Hepatosplenomegaly, Hepatomegaly, Liver, Children, Diagnostic delay, GBA1, Rare diseases

## Abstract

**Background:**

Neuronopathic Gaucher disease (GD types II and III) represents rare and severe phenotypes of glucocerebrosidase deficiency, characterized by neurological involvement and variable systemic manifestations. Data on clinical presentation and genotype–phenotype patterns in Eastern European populations remain limited.

**Methods:**

We conducted a retrospective cohort study of 92 patients with confirmed Gaucher disease followed at the National Children’s Specialized Hospital “Okhmatdyt” (Kyiv, Ukraine) between 2001 and 2024. Among them, 4 patients had type II and 6 had type III GD. Diagnosis was established by measurement of leukocyte β-glucocerebrosidase activity and molecular analysis of the *GBA1* gene. Clinical, laboratory, and imaging data were analyzed descriptively.

**Results:**

Type II GD presented in early infancy, with symptom onset from birth to 5 months of age (mean age at onset 2.8 ± 2.6 months) and was diagnosed between 5 and 9 months of age (mean 7.0 ± 2.3 months). Initial manifestations included cytopenias, splenomegaly, failure to thrive, and ichthyosiform dermatitis. Hepatomegaly was uncommon at onset, but hepatosplenomegaly was documented in all patients at diagnosis, followed by rapid neurological regression and death by 14 months of age. Type III GD had a mean age at onset of 2.3 ± 1.66 years and a mean age at diagnosis of 13.8 ± 16.87 years, reflecting substantial diagnostic delay. Oculomotor abnormalities were the most frequent presenting manifestations (83%). At diagnosis, all patients exhibited hepatosplenomegaly, cytopenias, and neurological involvement, while liver function remained preserved. Across both phenotypes, GBA1 variant combinations included genotypes predicted to result in very low or absent residual glucocerebrosidase activity, as well as genotypes predicted to retain some residual activity. Severe clinical courses were observed even in patients whose genotypes were predicted to retain residual enzyme activity, suggesting that predicted residual activity alone does not reliably explain clinical severity.

**Conclusions:**

In this Ukrainian cohort, neuronopathic GD demonstrated distinct clinical trajectories, with rapidly progressive infantile disease in type II and heterogeneous presentation with marked diagnostic delay in type III. Hepatosplenomegaly was a common systemic feature during disease progression, but should not be interpreted as an isolated stratifying sign. Its presence, particularly in combination with early or progressive neurological manifestations, should raise suspicion of neuronopathic GD.

## Introduction

Gaucher disease (GD; OMIM #230800, #230900, #231000) is a rare inherited metabolic disorder belonging to the group of lysosomal storage diseases. Its prevalence in the general population is estimated at 1 case per 60,000–100,000 live births. The disease is associated with a deficiency of the lysosomal enzyme glucocerebrosidase (EC 3.2.1.45). The resulting accumulation of glucosylceramide and related sphingolipids, predominantly within cells of the monocyte–macrophage system, leads to multisystem involvement, particularly affecting the spleen, liver, bone marrow, and skeletal system. The major systemic manifestations include hepatosplenomegaly, anaemia, thrombocytopenia, impaired growth, and skeletal abnormalities [1, 2]. Neuronopathic forms of the disease are considered separately, as their course is associated with involvement of the nervous system [3, 4].

Three major clinical phenotypes of GD are traditionally distinguished according to the presence, severity, and rate of progression of neurological involvement: type I (GD1; OMIM #230800), or non-neuronopathic GD; type II (GD2; OMIM #230900), or acute neuronopathic GD; and type III (GD3; OMIM #231000), or chronic neuronopathic GD [5]. However, these phenotypes represent a clinical continuum, and considerable variability may occur even among patients classified as having the same disease type.

Type II GD is the most severe neuronopathic form and is characterised by onset in infancy, rapidly progressive neurological deterioration, bulbar dysfunction, abnormalities of muscle tone, and severe visceral involvement. Additional manifestations may include ichthyosis, thrombocytopenia, developmental delay, dysphagia, and respiratory complications. The disease usually follows a fatal course during infancy or early childhood.

In contrast, type III GD is characterised by a more slowly progressive and clinically heterogeneous course. Its neurological manifestations may include horizontal supranuclear gaze palsy or impaired initiation of saccadic eye movements, oculomotor apraxia, ataxia, myoclonic or generalised epilepsy, cognitive impairment, and progressive motor dysfunction [3, 6]. In some patients, neurological abnormalities are subtle during the early stages of the disease and may become clinically evident only after systemic manifestations have already developed.

The clinical picture of type III GD also includes prominent visceral and haematological abnormalities. Hepatomegaly and splenomegaly, frequently accompanied by anaemia and thrombocytopenia, are among the most common manifestations. Bone involvement, growth retardation, delayed puberty, and pulmonary abnormalities may also occur. The combination and severity of these findings vary substantially between patients and may change during the course of the disease.

Enzyme replacement therapy (ERT) is the standard disease-specific treatment for Gaucher disease and is based on the administration of recombinant enzymes that functionally replace deficient β-glucocerebrosidase [7, 8]. Its main aim is to reduce glucocerebroside accumulation within lysosomes and to improve the systemic manifestations of the disease.

ERT is considered the gold standard for the treatment of systemic manifestations of Gaucher disease, including hepatosplenomegaly, anaemia, thrombocytopenia, and skeletal involvement. However, a major limitation of these therapies is their large molecular size, which prevents them from crossing the blood–brain barrier [9].

For this reason, in type II Gaucher disease, which is characterised by rapidly progressive central nervous system involvement, ERT is not considered a disease-modifying treatment. At present, there is no effective pathogenetic therapy for this form, and management is mainly symptomatic and palliative [7, 8].

However, in selected cases, ERT may be used temporarily to reduce visceral disease burden, cytopenias, or hepatosplenomegaly, with the understanding that it does not halt central nervous system progression or prevent the fatal neurodegenerative course of the disease.

Palliative and supportive care aims to improve quality of life and may include seizure control, respiratory support, nutritional support, physical therapy, and other symptomatic measures.

Type III GD progresses more slowly and is associated with variable neurological manifestations. Therefore, in this form, ERT is the standard of care and is effective in eliminating or reducing somatic manifestations, such as hepatosplenomegaly and hematologic abnormalities, but it does not affect neurological symptoms because of its limited ability to cross the blood-brain barrier [1].

In patients with type III GD, hematopoietic stem cell transplantation has been explored as a potential treatment option, particularly when the disease is diagnosed at the presymptomatic stage or early in the course of clinical manifestations. However, its use remains limited because of procedure-related risks, and available long-term data suggest that neurological manifestations may continue to progress after transplantation. Therefore, HSCT is not considered standard routine therapy for type III GD and should be considered only in selected cases in specialised centres [10].

Importantly, hepatosplenomegaly may be present before characteristic neurological manifestations become apparent. In young children, particularly those with mild or initially unrecognised abnormalities of eye movements, unexplained enlargement of the liver and spleen may therefore represent one of the earliest clinically detectable manifestations of type III GD. Nevertheless, because hepatosplenomegaly is a nonspecific finding associated with a broad range of haematological, infectious, malignant, and metabolic disorders, its diagnostic significance should be considered in combination with cytopenias, skeletal abnormalities, neurological findings, and the overall clinical course.

Early recognition of type III GD remains particularly important because enzyme replacement therapy (ERT) can effectively reduce hepatosplenomegaly and improve haematological and other systemic manifestations. However, currently available recombinant enzymes do not adequately cross the blood–brain barrier and therefore have little or no effect on the progression of neurological disease [1, 7, 8]. Consequently, delayed diagnosis may allow both systemic complications and neurological impairment to progress before disease-specific treatment and appropriate multidisciplinary care are initiated.

The aim of this study was to characterise the clinical manifestations and *GBA1* genotypes of Ukrainian patients with neuronopathic Gaucher disease, with particular emphasis on type II GD and type III GD. We specifically evaluated the frequency and timing of hepatosplenomegaly in relation to other systemic and neurological manifestations and assessed its potential value as an early clinical indicator of type III GD.

## Materials and methods

This retrospective study was conducted based on the analysis of medical records of 92 patients from different regions of Ukraine who were diagnosed with Gaucher disease between 2001 and 2024. The data were obtained from the Center for Orphan Diseases and Gene Therapy of the National Children’s Specialized Hospital “Okhmatdyt” of the Ministry of Health of Ukraine.

Diagnosis of the disease was established using biochemical and molecular genetic methods. In all patients, the activity of β-glucocerebrosidase in leukocytes (EC 3.2.1.45) and the plasma chitotriosidase (EC 3.2.1.14) level were determined. The reference range for leukocyte β-glucocerebrosidase activity was 5.1–9.5 nmol/h/mg protein, and the reference range for chitotriosidase was 0–159 nmol/h/mL plasma. Molecular genetic confirmation of the diagnosis was performed by analysis of allelic variants of the *GBA1* gene using Sanger sequencing and next-generation sequencing (NGS). Given the high sequence homology between the *GBA1* gene and its nearby pseudogene *GBAP1*, particular attention was paid to preventing pseudogene co-amplification and related sequencing artifacts. A nested PCR approach was performed prior to sequencing to ensure preferential amplification of the target *GBA1* regions and to minimize the risk of pseudogene-derived sequence interference and false-positive variant calls.

Clinical evaluation of the patients included detailed history taking, general physical examination, as well as instrumental and laboratory assessment of liver involvement. Ultrasonography (US) was used to assess liver size and structure, and functional status was evaluated based on serum transaminase levels. Liver and spleen size were considered enlarged if their volume exceeded the expected body weight-adjusted values by 2.5-fold or was more than 1.25-fold above the age-specific norm. Depending on the degree of liver and spleen enlargement, all patients were divided into four groups: “normal size” (within the age-specific norm), “mild enlargement” (up to 1.25-fold above the age-specific norm), “marked enlargement” (more than 1.25-fold but less than 2.0-fold above the age-specific norm), and “severe enlargement” (more than 2.0-fold above the expected age-specific value).

Clinical classification was applied retrospectively based on available medical records, age at symptom onset, neurological findings, disease course, and follow-up data. Patients were classified as type I GD when Gaucher disease was confirmed biochemically and/or genetically and no neurological manifestations compatible with neuronopathic Gaucher disease were documented during follow-up.

Type II GD was defined by onset in early infancy with rapidly progressive neurological involvement and a severe acute course. Type III GD was defined as chronic neuronopathic Gaucher disease, based on confirmed Gaucher disease with neurological involvement and a chronic rather than acute infantile course, in accordance with published consensus criteria: ocular motor abnormalities, especially gaze palsy or impaired saccadic eye movements, other neurological manifestations such as seizures, myoclonus, ataxia, spasticity, bulbar symptoms, cognitive impairment, or progressive neurological decline [11].

Allelic variants were classified according to the functional activity of β-glucocerebrosidase on the basis of residual enzyme activity: R (residual), referring to variants with preserved residual activity, and O (other/null), referring to variants leading to severe enzyme deficiency.

Statistical analysis was primarily descriptive due to the small sample size of neuronopathic cases. Continuous variables are presented as means ± standard deviation (SD) and ranges, while categorical variables are reported as absolute numbers and percentages. No inferential statistical comparisons between phenotypes were performed because of the limited number of patients in each subgroup. Data analysis was conducted using Statistica 7.0 (StatSoft Inc., Tulsa, OK, USA), MedCalc (MedCalc Software Ltd., Belgium), and Microsoft Excel.

## Results

**Fig. 1:**
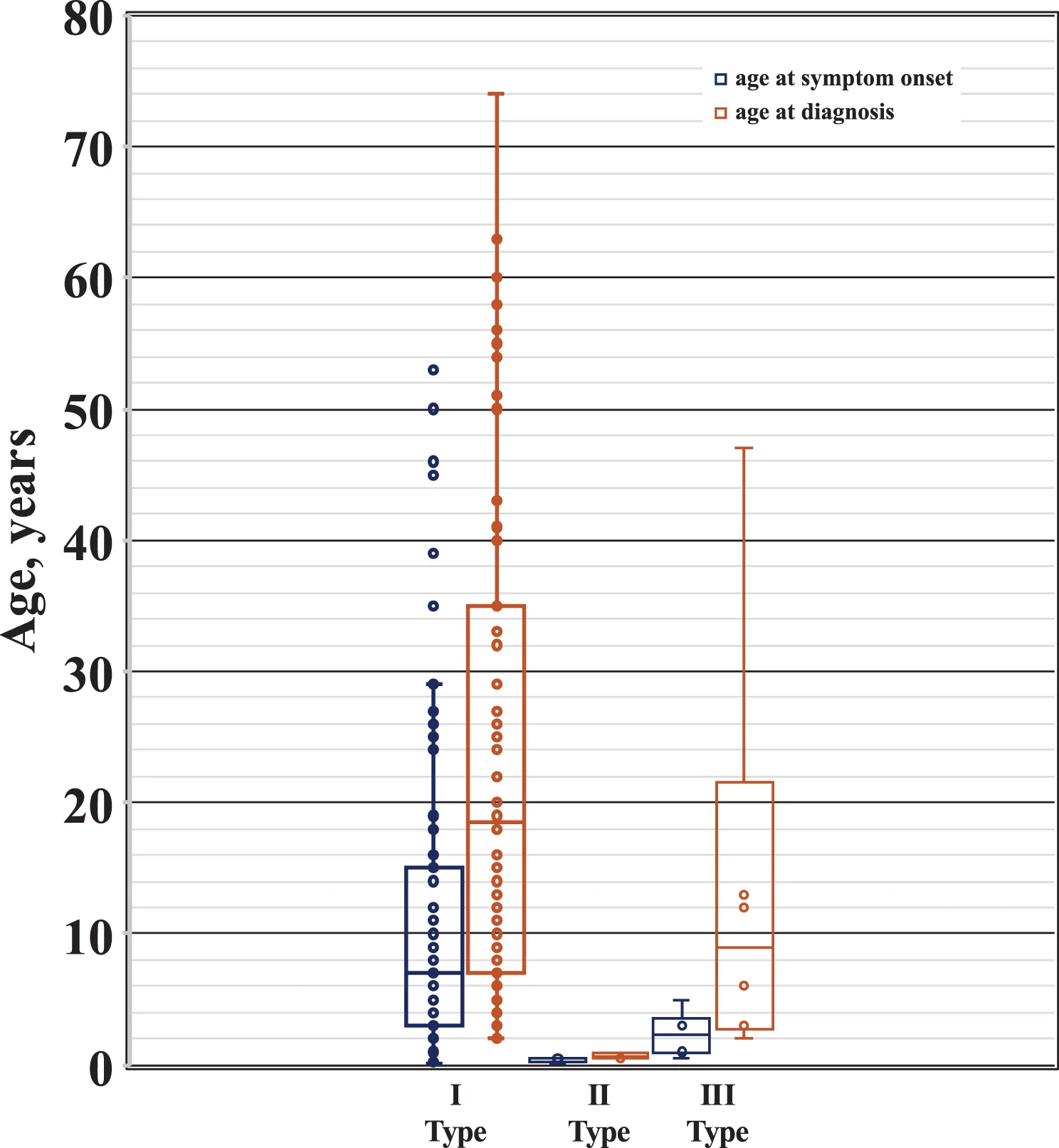
Age characteristics of patients with different types of GD

We analyzed the primary medical records of 92 patients (55 females and 37 males) who were diagnosed with Gaucher disease at the National Children’s Specialized Hospital “Okhmatdyt” between 2001 and 2024. Of these, 82 patients (89%) had type I GD, 4 children (4%) had type II GD, and 6 patients (7%) had type III GD (Fig. 1).

In patients with type II GD, the mean age at disease onset was 2.8 ± 2.6 months (range, birth to 5 months), and the mean age at diagnosis was 7.0 ± 2.3 months (range, 5–9 months).

Patients with type II GD were diagnosed relatively quickly, with a mean interval of 4.3 ± 3.7 months between symptom onset and diagnosis (range, 0–9 months).

**Fig. 2:**
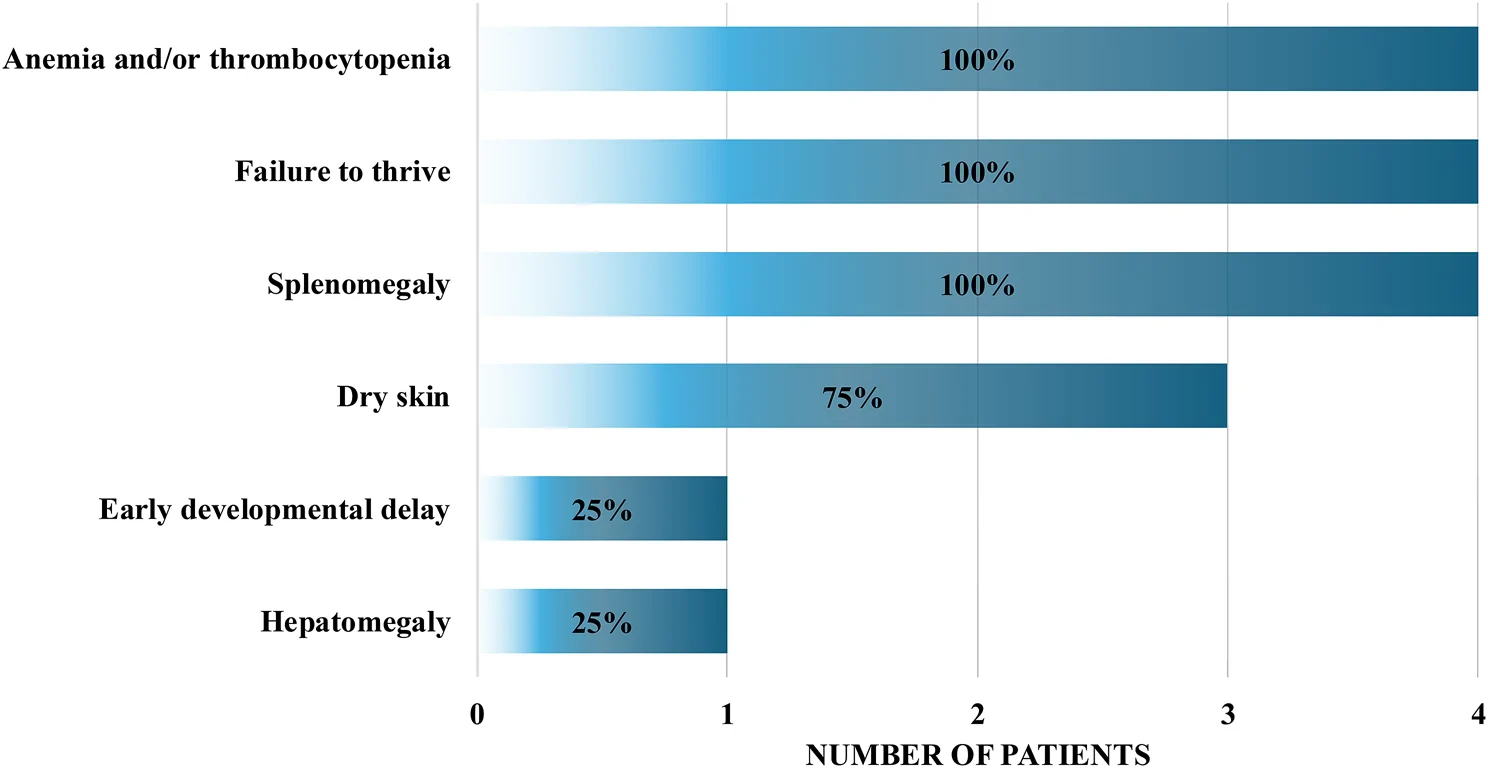
Distribution of presenting symptoms in patients with type II GD

In all patients, type II GD presented with hematologic abnormalities, splenomegaly, and failure to thrive. In one child (25%), splenomegaly was accompanied by hepatomegaly. Three of the four children (75%) had dry skin from birth, which was interpreted as ichthyosiform dermatitis. One patient (25%) also had early developmental delay, and one patient had scrotal edema (Fig. 2).

At presentation to the National Children’s Specialized Hospital “Okhmatdyt”, all patients were in severe condition, with delayed or regressing motor development, ichthyosis, failure to thrive, and marked hepatosplenomegaly. These symptoms developed rapidly, within 2–3 months of disease onset.

**Fig. 3:**
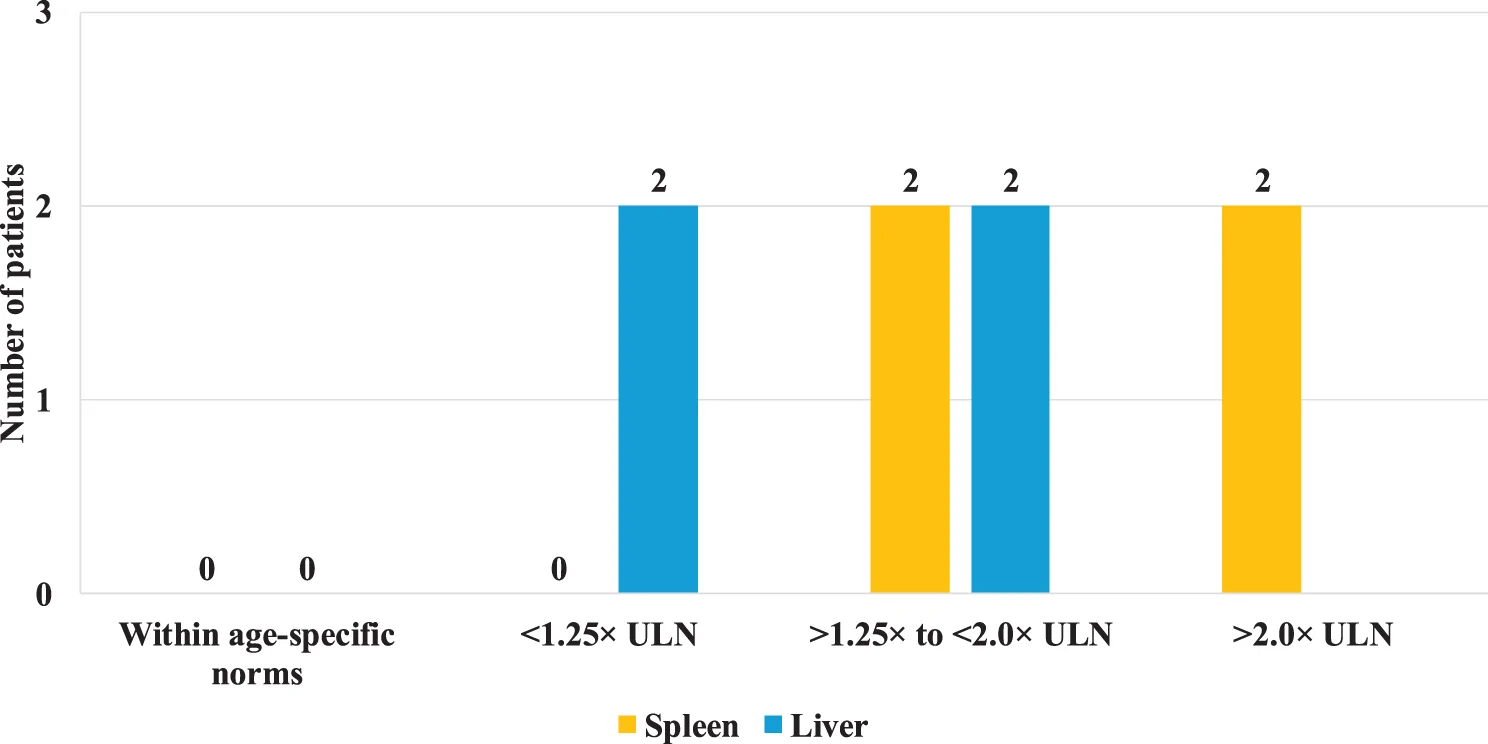
Hepatomegaly and splenomegaly at initial diagnosis in patients with type II GD at the National Children’s Specialized Hospital “Okhmatdyt”

At the time of diagnosis, all children had hepatosplenomegaly of varying severity. Two patients (50%) had mild hepatomegaly, with liver size up to 1.25-fold above the age-specific norm, whereas in the remaining two patients (50%) hepatomegaly was more pronounced, ranging from >1.25-fold to <2.0-fold above the age-specific norm. Splenomegaly was more severe: in two children (50%), spleen size exceeded the age-specific norm by >1.25-fold to <2.0-fold, and in the other two children (50%), by 2.0-fold (Fig. 3).

All patients (4/4) showed biochemical evidence of hepatocellular injury, with serum aminotransferase levels approximately 2–3 times the upper limit of normal. All children also showed substantial reductions in platelet counts and hemoglobin levels. Two patients developed severe portal hypertension and liver failure. All four patients developed neurological involvement characterized by developmental delay or regression. Opisthotonus and bulbar or pseudobulbar dysfunction, including swallowing difficulties and choking or aspiration episodes, were documented in three patients (75%). Abnormal eye movements, specifically supranuclear gaze palsy, were documented in two patients. All four patients developed neurological involvement characterized by developmental delay or regression. Opisthotonus and bulbar or pseudobulbar dysfunction, including swallowing difficulties and choking or aspiration episodes, were documented in three patients (75%). Seizures were also documented in three patients (75%; Patients 2–4), with onset between 8 and 9 months of age. Abnormal eye movements, specifically supranuclear gaze palsy, were documented in two patients (Table 1).

**Table 1:** Genotypes and major clinical features observed in patients with type II GD

	Patients			
	1	2	3	4
Sex	F	M	M	F
Age at onset (months)	0	1	5	5
Age at diagnosis (months)	9	5	9	5
Outcome, Age at death (months)	10	14	13	10
Chitotriosidase activity (nmol/h/mL plasma)	ND	6000	12906	7956
β-Glucocerebrosidase activity (nmol/h/mg protein)	ND	2.2	3.2	3.3
GBA1 genotype (cDNA)	c.475C > T /c.255_257delGCG	c.1288C > G /c.84dup	c.721 G > A /c.1268C > A	c.721 G > A /c.604C > T
Functional classification	R/O	R/O	R/O	O/O
Presenting symptoms	Ichthyosis, anemia, splenomegaly, scrotal edema	Ichthyosis, hepatosplenomegaly, failure to thrive	Anemia, failure to thrive, ichthyosis	Failure to thrive, hepatosplenomegaly, developmental delay
**Maximum documented severity during natural disease course**				
scrotal edema	++	+	ND	ND
Ichthyosis	++	++	ND	ND
Failure to thrive	+++	+++	+++	+++
Bulbar / pseudobulbar signs	ND	++	++	++
Choking or aspiration episodes	+++	+	ND	++
Stridor	+	ND	ND	+
Swallowing difficulties	+	+	++	++
Hypertonia	ND	++	+++	+
Respiratory compromise	++	ND	+	+
Hepatomegaly	+++	+++	+++	++
Splenomegaly	+++	+++	+++	+++
Developmental delay or regression	+++	+++	+++	+++
Opisthotonus	ND	+	+	++
Seizures	ND	++	++	++
Abnormal eye movements	ND	+	++	ND
Cytopenias:				
anemia	+++	+++	+++	+++
thrombocytopenia	+++	+++	+++	+++
Liver dysfunction	++	+++	++	+

Manifestation severity was graded retrospectively from the available medical records: +, mild manifestation without major functional impact; ++, clinically significant manifestation requiring monitoring or supportive care; +++, severe, progressive, or life-threatening manifestation and/or requiring major intervention; ND, not documented or not assessed

**Fig. 4:**
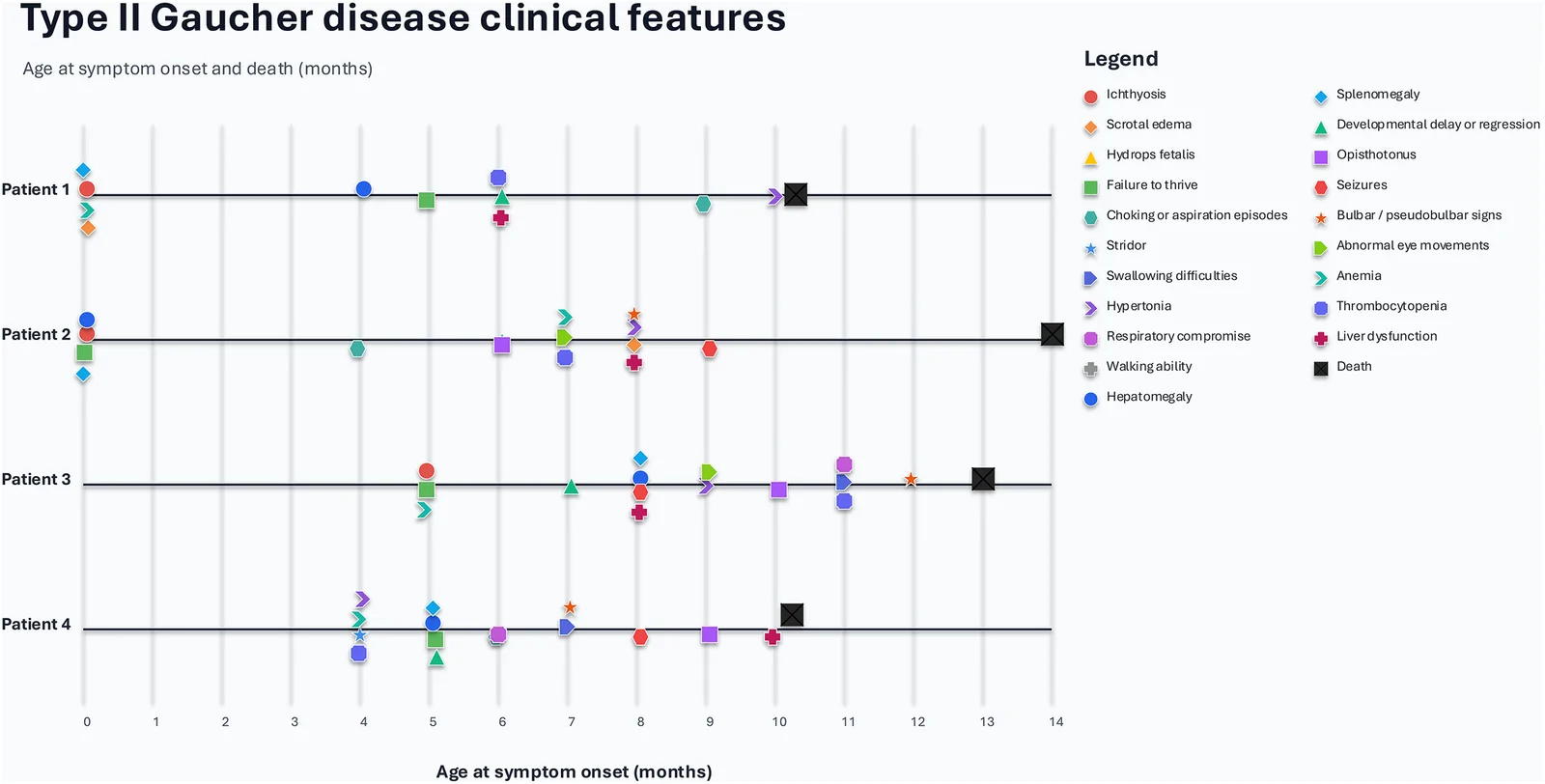
Temporal distribution of clinical manifestations in four patients with type II Gaucher disease

The temporal evolution of clinical manifestations in the four patients with type II GD is shown in Fig. 4. The earliest manifestations occurred during the first months of life and included ichthyosis, scrotal edema, anemia, and splenomegaly. Failure to thrive developed in all patients by 5 months of age. Hepatomegaly and splenomegaly were documented in all four patients between birth and 8 months of age. Neurological involvement also developed early: developmental delay or regression was observed between 5 and 7 months, hypertonia between 4 and 10 months, and opisthotonus in three patients between 6 and 10 months. Neurological involvement also developed early: developmental delay or regression was observed between 5 and 7 months, hypertonia between 4 and 10 months, and opisthotonus in three patients between 6 and 10 months. Seizures were documented in Patients 2–4 between 8 and 9 months of age. Bulbar dysfunction, including swallowing difficulties and choking or aspiration episodes, was documented in three patients, while respiratory compromise occurred in two patients at 6 and 11 months, respectively. Abnormal eye movements were reported in two patients. All four patients died between 10 and 14 months of age.

All patients underwent comprehensive molecular genetic testing. Pathogenic variants in the *GBA1* gene were identified, including c.1288C > G/c.84dup, c.721 G > A/c.1268C > A, c.721 G > A/c.604C > T, and c.255_257delGCG/c.475C > T (Table 1).

According to the classification of *GBA1* variants based on their functional effect on residual enzyme activity, c.1288C > G, c.1268C > A, and c.475C > T were classified as R variants (residual-function variants), whereas c.84dup, c.721 G > A, c.604C > T, and c.255_257delGCG were classified as O variants (severe/null variants) [12–15]. Accordingly, among patients with type II GD, we identified the following R/O combinations: c.475C > T/c.255_257delGCG, c.1288C > G/c.84dup, and c.1268C > A/c.721 G > A, as well as one severe O/O combination: c.721 G > A/c.604C > T.

In three patients, chitotriosidase activity exceeded the reference values by 40- to 86-fold, with a mean increase of 59.7 ± 23.5-fold. In the same patients, β-glucocerebrosidase activity ranged from 43% to 65% of the lower reference limit, with a mean of 56.8 ± 12.4%.

In one patient, lysosomal enzyme activity could not be determined because of extensive prior blood transfusions.

In type III GD, the mean age at disease onset was 2.3 ± 1.66 years (range, 0.5–5 years), and the mean age at diagnosis was 13.8 ± 16.87 years (range, 2–47 years) (Fig. 1).

**Fig. 5:**
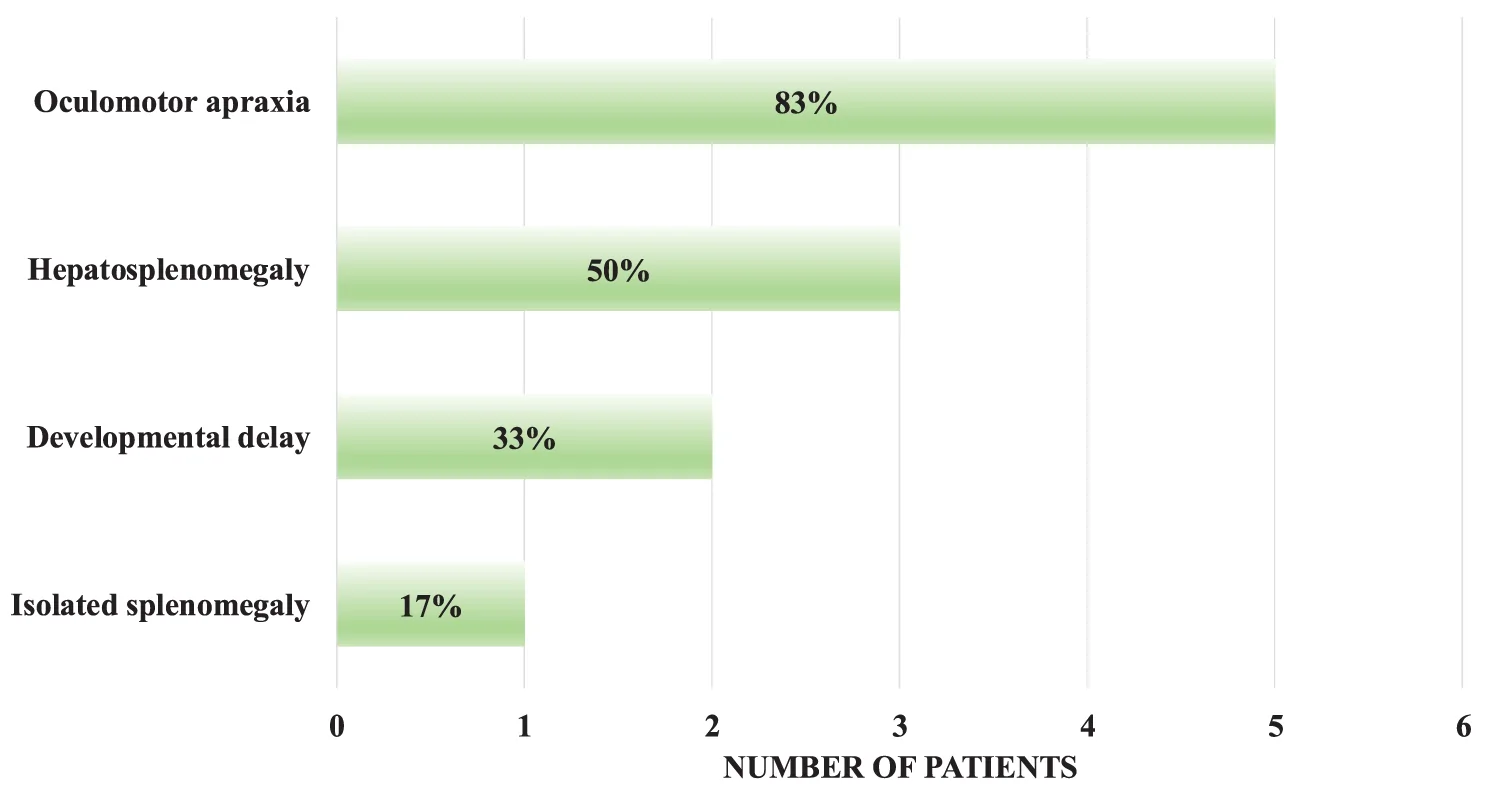
Distribution of symptoms at disease onset in patients with type III GD

Among the six patients with type III GD, the most common initial manifestation was abnormal horizontal saccades or supranuclear gaze palsy, observed in five patients (83%). Hepatosplenomegaly was present at disease onset in three patients (50%), developmental delay in two patients (33%), and isolated splenomegaly in one patient (17%) (Fig. 5).

The mean interval from the onset of the first symptoms to diagnosis was 11.5 ± 17.5 years (range, 1–46.5 years). At the time of diagnosis, all patients had neurological manifestations accompanied by thrombocytopenia, anemia, marked splenomegaly, and hepatomegaly, without biochemical evidence of impaired liver function.

During the natural course of the disease, ataxia was documented in five patients. Myoclonus was documented in five patients, cognitive impairment in four, seizures in four, dysarthria in four, and tremor in two. Abnormal horizontal saccades or supranuclear gaze palsy were documented in five patients. Four patients died between 10.5 and 15 years of age. In all four deceased patients, death was associated with respiratory complications in the context of progressive neurological deterioration.

Patient 6 had no documented abnormality of horizontal saccades or supranuclear gaze palsy. Nevertheless, he developed a slowly progressive neurological phenotype characterized by ataxia, myoclonus, mild cognitive impairment, dysarthria, and lower-limb paraparesis. MRI revealed spinal cord myelopathy; however, its relationship to the primary neuronopathic process or to secondary skeletal complications could not be definitively established. Based primarily on the chronic progressive neurological manifestations in the setting of enzymatically and genetically confirmed GD, the patient was classified as having type III GD.

**Fig. 6:**
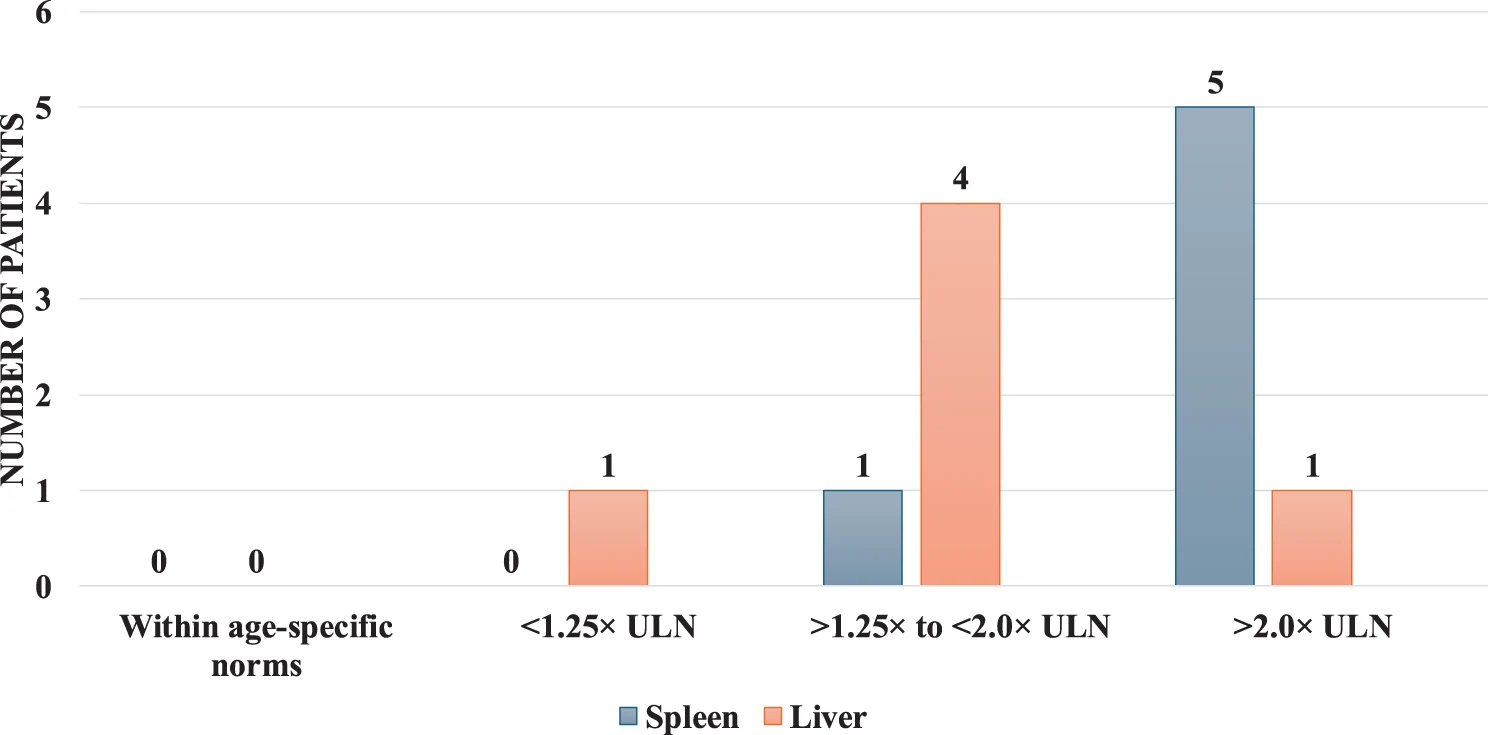
Hepatomegaly and splenomegaly at initial diagnosis in patients with type III GD at the National Children’s Specialized Hospital “Okhmatdyt”

Hepatomegaly was present in all patients. One child (17%) had mild hepatomegaly, with liver size up to 1.25-fold above the age-specific norm; in four patients (67%), hepatomegaly was more pronounced, ranging from >1.25-fold to <2.0-fold above the age-specific norm; and in one patient (17%), liver size exceeded the age-specific norm by more than 2.0-fold. Splenomegaly was markedly more severe in all patients: in one patient (17%), spleen size exceeded the age-specific norm by >1.25-fold to <2.0-fold, whereas in the remaining five patients (83%), it exceeded the age-specific norm by more than 2.0-fold (Fig. 6).

Transaminase and bilirubin levels in all patients remained within the normal range. According to ultrasound examinations, splenic infarction was detected in five patients, whereas one patient had fibrotic liver changes and calculous cholecystitis.

**Fig. 7:**
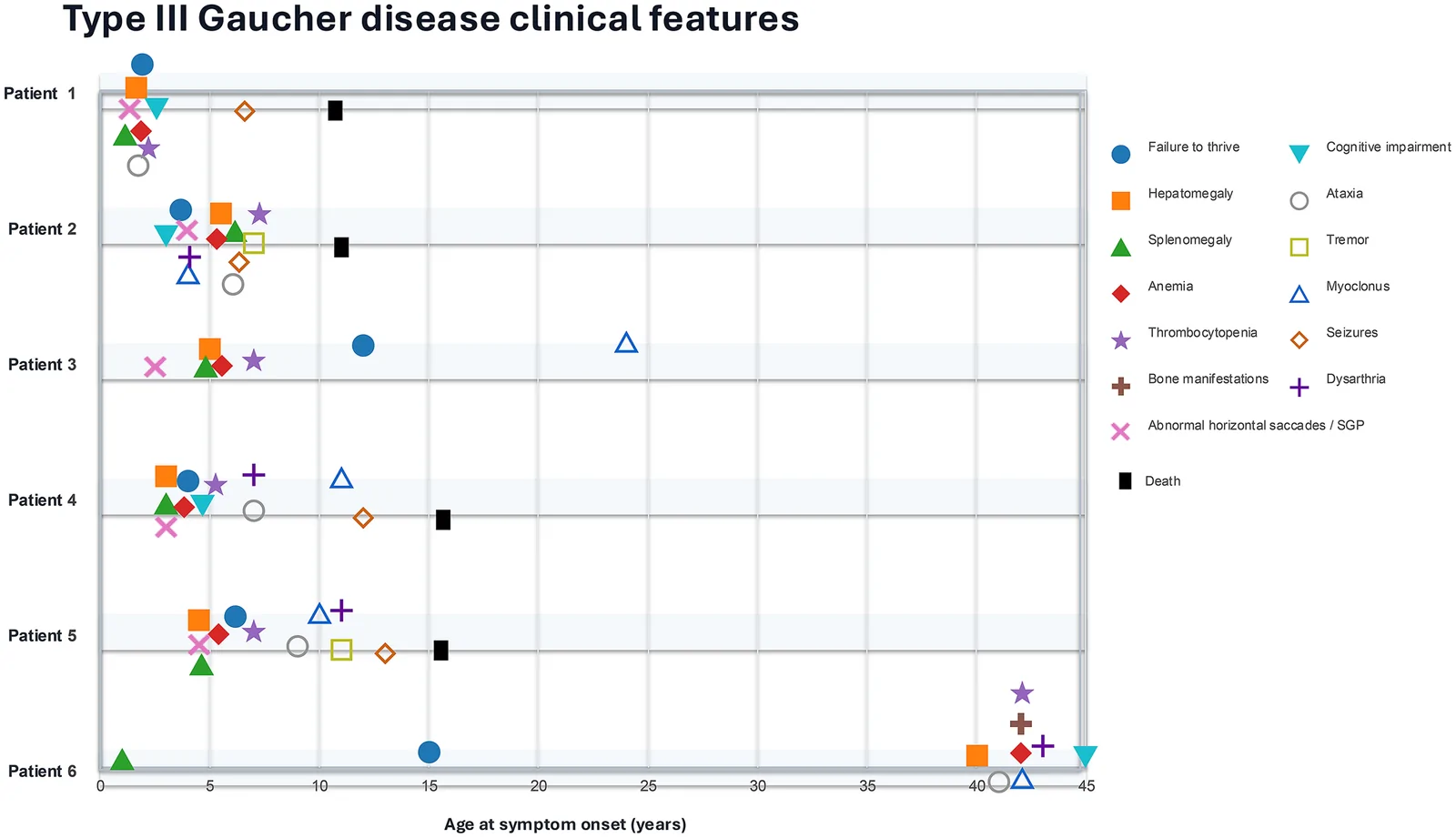
Temporal distribution of clinical manifestations in six patients with type III Gaucher disease

The temporal distribution of clinical manifestations in the six patients with type III GD is shown in Fig. 7. In five patients, visceral, hematological, and oculomotor manifestations developed during early childhood. Hepatomegaly and splenomegaly were generally documented between 1 and 5.5 years of age, while anemia and thrombocytopenia usually appeared within the first 2–7 years of life. Abnormal horizontal saccades or supranuclear gaze palsy were recorded in five patients, with onset between approximately 1.3 and 4.5 years. Neurological manifestations subsequently progressed and included ataxia in five patients, myoclonus in five, cognitive impairment in four, seizures in four, dysarthria in four, and tremor in two. The timing and sequence of neurological progression varied considerably between patients. Patient 6 showed a distinctly slower course, with splenomegaly noted in infancy but most neurological, hematological, and skeletal manifestations developing only in adulthood. Severe skeletal involvement was documented in this patient at 42 years of age and included recurrent bone crises, osteonecrosis, and osteopenia.

All patients underwent comprehensive molecular genetic testing. Pathogenic variants in the *GBA1* gene were identified, including c.721 G > A, c.1448T > C, c.203dupC, c.1246 G > A, c.1342 G > C, c.1129 G > A, c.999 G > A, c.680A > G, c.732C > A, and c.1226A > G (Table 2).

**Table 2:** Genotypes and major clinical features observed in patients with type III GD

Patients						
	1	2	3	4	5	6
Sex	M	F	M	M	M	M
Age at onset (years)	1.5	3	5	1	3	0.5
Age at diagnosis (years)	3	6	12	2	13	47
Age at death (years)	10.5	12	-	15	15	-
Chitotriosidase activity (nmol/h/mL plasma)	19448	7293	4552	11934	6632	10530
β-Glucocerebrosidase activity (nmol/h/mg protein)	1.6	2.6	4.2	2.9	2.2	1.6
GBA1 genotype (cDNA)	c.721 G > A/c.1448T > C	c.1246 G > A/c.203dupC	c.1342 G > C/c.721 G > A	c.1129 G > A/c.999 G > A	c.680A > G/c.732C > A	c.1226A > G/c.1448T > C
Functional classification	O/O	R/O	R/O	R/R	R/O	R/O
Presenting symptoms	Hepatosplenomegaly, gaze palsy, developmental delay	gaze palsy, developmental delay	gaze palsy	Hepatosplenomegaly, gaze palsy	Hepatosplenomegaly, gaze palsy	Splenomegaly
Maximum documented severity during natural disease course						
Failure to thrive	++	+++	+	+++	+++	+
Hepatomegaly	+++	+++	++	+++	+++	+
Splenomegaly	+++	+++	++	+++	+++	+++
Anemia	+++	+++	+	++	++	+
Thrombocytopenia	+++	+++	+	++	++	+
Bone manifestations	ND	ND	ND	ND	ND	+++
Abnormal horizontal saccades / supranuclear gaze palsy	+++	+++	+++	+++	+++	ND
Cognitive impairment	++	+++	ND	+++	ND	+
Ataxia	++	+++	+	+++	+++	+
Tremor	ND	+	ND	ND	+++	ND
Myoclonus	ND	+++	+	+++	+++	+
Seizures	+++	+++	ND	+++	++	ND
Dysarthria	ND	++	+	+	+	+

Manifestation severity was graded retrospectively from the available medical records: +, mild manifestation without major functional impact; ++, clinically significant manifestation requiring monitoring or supportive care; +++, severe, progressive, or life-threatening manifestation and/or requiring major intervention; ND, not documented or not assessed

According to the functional classification of *GBA1* variants based on their effect on residual enzyme activity, c.721 G > A, c.1448T > C, c.203dupC, and c.732C > A were classified as O variants (severe/null variants). Other variants, including c.1226A > G, c.1246 G > A, c.680A > G, c.1342 G > C, c.1129 G > A, and c.999 G > A, were classified as R variants (residual-function variants) [12–15]. Among Ukrainian patients, the following genotypes were identified: O/O - c.721 G > A/c.1448T > C; R/O - c.203dupC/c.1246 G > A, c.721 G > A/c.1342 G > C, c.680A > G/c.732C > A, and c.1226A > G/c.1448T > C; and R/R - c.1129 G > A/c.999 G > A.

Manifestation severity was graded retrospectively from the available medical records: +, mild manifestation without major functional impact; ++, clinically significant manifestation requiring monitoring or supportive care; +++, severe, progressive, or life-threatening manifestation and/or requiring major intervention; ND, not documented or not assessed

In the study patients, chitotriosidase activity exceeded the reference range by 30- to 130-fold, with a mean increase of 67.1 ± 32.8-fold. β-Glucocerebrosidase activity ranged from 31% to 82% of the lower reference limit, corresponding to a mean activity of 2.52 ± 0.93 nmol/mg/h, or 52.5 ± 19.4%.

## Discussion

Compared with type I GD, types II and III GD are rarer and less well-studied forms of Gaucher disease. Limited physician awareness of the neuronopathic course of GD often leads to delayed diagnosis. This may reduce the effectiveness of disease-specific therapy when available and may also result in unnecessary diagnostic procedures in affected children. Central nervous system involvement, which characterizes these phenotypes, should be taken into account in the differential diagnosis of patients with suspected lysosomal storage disorders (LSDs).

In this national series, comprehensive diagnostic evaluation of GD is currently performed only at the Laboratory of Medical Genetics of the National Children’s Specialized Hospital “Okhmatdyt.” Thus, this center allows analysis of all known diagnosed cases in the present study.

According to international GD registries, type II accounts for approximately 1% of all reported GD cases, whereas type III accounts for about 5% [16, 17]. In the Ukrainian cohort, according to our own data, type II GD and type III GD accounted for 4 and 7% of cases, respectively. This difference is most likely related to the small sample size, physicians awareness of the clinical features of neuronopathic GD, and the availability of diagnostic testing for this disorder.

The course of type II GD in Ukrainian patients generally corresponds to previously published data reported by other authors [3, 5, 18].

For comparison of the type II GD cohort, we primarily considered European data, particularly the French series reported by Mignot et al. [19], which included 15 patients with acute neuronopathic Gaucher disease. Similar to that cohort, all four Ukrainian patients developed symptoms during infancy and showed rapidly progressive neurological disease characterized by developmental delay or regression, hypertonia, opisthotonus, and bulbar dysfunction. Visceral and hematological involvement was also prominent, with hepatosplenomegaly, anemia, and thrombocytopenia documented in all patients.

However, the Ukrainian patients showed particularly early and marked systemic involvement. In some patients, ichthyosis, scrotal edema, anemia, and splenomegaly were already present during the first weeks of life. All patients subsequently developed biochemical evidence of hepatocellular injury, and two developed severe portal hypertension and liver failure. These findings suggest a substantial visceral and hepatic disease burden in addition to the rapidly progressive neurological phenotype.

Hepatosplenomegaly is a common feature in patients with type II GD. All children included in the study showed enlargement of both the liver and spleen. Hepatomegaly was mild in half of the patients (50%), with liver size up to 1.25-fold above the expected age-specific norm, whereas in the remaining 50% it was more pronounced, ranging from >1.25-fold to <2.0-fold above the age-specific norm. Splenomegaly was more severe: most children showed spleen enlargement in the ranges of >1.25-fold to <2.0-fold and more than 2.0-fold above the age-specific norm. These findings are consistent with published data [1, 3] and may represent a marker of severe disease course and rapid progression.

Using the recently proposed age- and phenotype-based classification of 250 type II GD cases [20], all four Ukrainian patients would be placed predominantly within the early infantile neurodegenerative category. Symptoms developed within the first six months of life and were followed by rapid neurological, bulbar, and respiratory deterioration. Nevertheless, the presence of ichthyosis, scrotal edema, early cytopenias, and hepatosplenomegaly during the neonatal period in some patients indicates partial overlap with the neonatal neurodegenerative or inflammatory presentations. This overlap supports the concept that acute neuronopathic GD comprises a phenotypic continuum rather than sharply separated clinical subgroups.

Overall, the Ukrainian type II GD cohort was consistent with the severe early infantile end of the acute neuronopathic Gaucher disease spectrum described in European series. However, it was distinguished by the coexistence of rapidly progressive neurological disease with marked visceral, hematological, and hepatic involvement. Hepatomegaly and splenomegaly were present in all four patients, appeared early in the disease course, and in some cases preceded overt neurological deterioration. Bulbar dysfunction and respiratory complications were prominent, and all four patients died between 10 and 14 months of age. The presence of different compound heterozygous GBA1 genotypes further emphasizes the genetic and phenotypic heterogeneity of type II GD in this population.

The clinical presentation of type III GD is substantially more heterogeneous than that of type II GD and is characterized by a more slowly progressive chronic neuronopathic course. In the Ukrainian cohort, the initial manifestations included oculomotor abnormalities, hepatosplenomegaly, and developmental delay. Hepatomegaly and splenomegaly were documented in all six patients and, in several cases, preceded the recognition of neurological involvement. Over time, the phenotype progressed to marked hepatosplenomegaly and neurological manifestations, including ataxia, myoclonus, seizures, dysarthria, and regression of cognitive and motor development. These findings are consistent with previously published data on the natural history of type III GD [6, 21]. Four patients died between 10.5 and 15 years of age from respiratory complications in the setting of progressive neurological deterioration. Notably, one patient had no documented abnormality of horizontal saccades or supranuclear gaze palsy despite a chronic progressive neurological course. This observation supports the view that the absence of characteristic oculomotor findings does not exclude type III GD when other progressive neurological manifestations are present.

For regional comparison, we considered the Polish cohort [22] because Poland is the geographically closest country with a detailed national description of neuronopathic GD. None of the Ukrainian patients exhibited the Norrbottnian-like phenotype reported in the Polish cohort, which was characterized by early massive visceral disease, progressive kyphoscoliosis, relatively mild cognitive impairment, and survival for at least three decades. Instead, most Ukrainian patients more closely resembled the severe progressive neurological Polish phenotype, particularly because of the prominent myoclonus, ataxia, seizures, cognitive impairment, and absence of characteristic progressive kyphoscoliosis. Nevertheless, the Ukrainian cohort differed from this Polish subgroup by the coexistence of severe neurological progression with pronounced visceral and hematological involvement. The marked differences in both phenotype and GBA1 genotype distribution are notable given the geographical proximity of Ukraine and Poland and suggest substantial regional heterogeneity of neuronopathic GD within Eastern Europe.

Peripheral neuropathy does not appear to be a characteristic neurological manifestation of type III GD. In a Swedish cohort, Andréasson et al. found no neurophysiological evidence of small- or large-fiber polyneuropathy attributable to of type III GD [23].

Splenomegaly and hepatomegaly are consistent clinical features in patients with type III GD. As in type II GD, all patients exhibited hepatosplenomegaly. Hepatomegaly was mild in only one patient (17%), with liver size up to 1.25-fold above the expected age-specific norm, whereas most patients (67%) had more pronounced enlargement in the range of >1.25-fold to <2.0-fold, and in one patient (17%) liver size exceeded the age-specific norm by more than 2.0-fold. Splenomegaly was more pronounced: most patients showed spleen enlargement in the ranges of >1.25-fold to <2.0-fold and more than 2.0-fold above the age-specific norm. These observations are supported by other clinical data indicating that, in patients with type III GD, splenomegaly may be more pronounced than hepatomegaly [21].

Analysis of chitotriosidase activity, which was markedly elevated above the reference range in patients with both type II and type III GD, indicates pronounced macrophage activation in response to glucocerebroside accumulation [24]. At the same time, residual β-glucocerebrosidase activity was preserved in these patients, although this did not prevent the development of a severe clinical phenotype [14, 15].

Among the pathogenic *GBA1* variants analyzed in patients with type II and type III GD, R/R, R/O, and O/O combinations were identified according to the classification based on residual enzyme activity. In patients carrying variants associated with potential residual activity (R), the clinical course was nevertheless severe, with early disease onset and rapid progression, which is characteristic of the acute and chronic neuronopathic forms of GD. This is consistent with published data suggesting that even R/O combinations may lead to severe forms of the disease because of the dominant negative effect of the null variant [21, 25, 26]. Other disease-modifying factors should also be considered. These include the accumulation of secondary metabolites, disruption of calcium (Ca^2 +^) homeostasis, oxidative stress, chronic inflammation, abnormalities of lipid transport, impaired autophagy, endoplasmic reticulum (ER) stress, and activation of the unfolded protein response [25, 27].

Delayed diagnosis of neuronopathic Gaucher disease has particularly serious consequences because the disease continues to progress before specific therapy is initiated, and some of the resulting changes become irreversible. Late treatment initiation contributes to worsening hepatosplenomegaly, cytopenias, skeletal disease, and other systemic complications, while established neurological deficits—including oculomotor abnormalities, ataxia, myoclonus, and cognitive and motor deterioration—are generally not reversible. Enzyme replacement therapy can effectively control visceral and hematological manifestations; however, it does not cross the blood–brain barrier and therefore does not halt progression of central nervous system involvement. Early diagnosis is thus critical not only for timely initiation of ERT, but also for limiting the accumulation of irreversible organ and neurological damage. In Ukraine, ERT for patients with Gaucher disease is state-funded and procured centrally, although actual access depends on timely diagnostic confirmation, inclusion in the treatment program, continuity of procurement, and reliable supply logistics.

To our knowledge, this study represents the first systematic national analysis of neuronopathic Gaucher disease in Ukraine, encompassing all diagnosed cases managed at the country’s primary referral center over more than two decades. The findings demonstrate distinct clinical trajectories, with rapidly progressive infantile disease in type II GD and a more heterogeneous, chronically progressive neurological phenotype in type III GD. In particular, type III GD was associated with a striking diagnostic delay, with a mean interval exceeding a decade between symptom onset and diagnosis, underscoring the need for greater awareness among pediatricians, neurologists, and other specialists. Hepatosplenomegaly was a consistent systemic feature during disease progression in both phenotypes, but should not be interpreted as an isolated stratifying sign. Rather, its presence, particularly in combination with early or progressive neurological manifestations such as oculomotor abnormalities, developmental regression, ataxia, myoclonus, seizures, or bulbar dysfunction, should raise suspicion of neuronopathic GD and prompt specific enzymatic and molecular testing. Furthermore, the observed clinical severity in patients carrying variants predicted to retain residual enzyme activity reinforces the limited predictive value of genotype–phenotype correlations in this cohort and suggests that additional genetic, epigenetic, or environmental modifiers may influence disease expression.

## Limitations

This study has several limitations. First, the sample size of patients with neuronopathic Gaucher disease was small, reflecting the rarity of these phenotypes and limiting the ability to perform inferential statistical analyses. Second, the retrospective design may have resulted in incomplete documentation of early clinical manifestations and variability in follow-up data. Third, the study was conducted at a single national referral center, which may introduce referral bias and limit the generalizability of findings to other populations. Finally, although genetic variants were classified according to previously published data on predicted residual enzyme activity, genotype–phenotype correlations remain inherently complex in neuronopathic Gaucher disease and should be interpreted with caution.

## Conclusion

This study provides the first systematic national description of neuronopathic Gaucher disease in Ukraine and demonstrates distinct clinical trajectories for types II and III GD. Type II GD followed a rapidly progressive infantile course with early mortality, whereas type III GD showed marked phenotypic heterogeneity and substantial diagnostic delay. Hepatosplenomegaly was a common systemic feature in both phenotypes but should be interpreted in conjunction with neurological manifestations rather than as an isolated stratifying sign. Early recognition of oculomotor abnormalities, developmental regression, ataxia, myoclonus, seizures, and bulbar dysfunction in patients with hepatosplenomegaly should prompt enzymatic and molecular testing for neuronopathic GD. The observed clinical variability despite predicted residual enzyme activity further supports the limited value of genotype alone for prognostic assessment. These findings may help improve diagnostic pathways, specialist awareness, and national registry development in Ukraine.

## Data Availability

The datasets generated and analysed during the current study are not publicly available due to patient privacy and ethical restrictions, but are available from the corresponding author on reasonable request.
